# The M50I polymorphic substitution in association with the R263K mutation in HIV-1 subtype B integrase increases drug resistance but does not restore viral replicative fitness

**DOI:** 10.1186/1742-4690-11-7

**Published:** 2014-01-17

**Authors:** Melissa Wares, Thibault Mesplède, Peter K Quashie, Nathan Osman, Yingshan Han, Mark A Wainberg

**Affiliations:** 1 Lady Davis Institute for Medical Research, McGill University AIDS Centre, Jewish General Hospital, Montreal, Quebec, Canada; 2 Department of Microbiology and Immunology, Faculty of Medicine, McGill University, Montreal, Quebec, Canada; 3 Division of Experimental Medicine, Faculty of Medicine, McGill University, Montreal, Quebec, Canada

**Keywords:** HIV integrase, Subtype B, Antiretrovirals, R263K, Resistance mutation, M50I, Polymorphism, INSTI-naïve

## Abstract

**Background:**

First-generation integrase strand-transfer inhibitors (INSTIs), such as raltegravir (RAL) and elvitegravir (EVG), have been clinically proven to be effective antiretrovirals for the treatment of HIV-positive patients. However, their relatively low genetic barrier for resistance makes them susceptible to the emergence of drug resistance mutations. In contrast, dolutegravir (DTG) is a newer INSTI that appears to have a high genetic barrier to resistance *in vivo*. However, the emergence of the resistance mutation R263K followed by the polymorphic substitution M50I has been observed in cell culture. The M50I polymorphism is also observed in 10-25% of INSTI-naïve patients and has been reported in combination with R263K in a patient failing treatment with RAL.

**Results:**

Using biochemical cell-free strand-transfer assays and resistance assays in TZM-bl cells, we demonstrate that the M50I polymorphism in combination with R263K increases resistance to DTG in tissue culture and in biochemical assays but does not restore the viral fitness cost associated with the R263K mutation.

**Conclusions:**

Since the combination of the R263K mutation and the M50I polymorphism results in a virus with decreased viral fitness and limited cross-resistance, the R263K resistance pathway may represent an evolutionary dead-end. Although this hypothesis has not yet been proven, it may be more advantageous to treat HIV-positive individuals with DTG in first-line than in second or third-line therapy.

## Background

The development of effective antiretrovirals against HIV has led to significant improvements in longevity and quality of life for HIV-infected patients. Despite the progress made in the past 30 years, however, significant issues with HIV treatment remain including those related to drug resistance, tolerability, ease of dosing, and adherence to therapy [1]. Consequently, new targets for HIV are required to overcome problems associated with current antiretroviral (ARV) drug classes. The most recently developed drug class is integrase-strand transfer inhibitors (INSTIs) that inhibit the previously untargeted HIV-1 integrase enzyme [2,3]. HIV-1 integrase (IN) is a protein comprising 288 amino acids structured into three domains, i.e. the N-terminal domain, the catalytic core domain and the C-terminal domain [4]. These domains are critical for the proper function of HIV-1 integrase, which is to catalyze the insertion of the proviral DNA into the host chromosome. This process is achieved by two reactions. The first reaction, referred to as 3′ processing, is defined as the cleavage of a GT dinucleotide at the 3′ ends of the viral DNA resulting in the exposure of reactive hydroxyl groups [5]. These 3′ hydroxyl groups then serve to covalently link the processed viral DNA and the host DNA in a reaction referred to as strand-transfer [5]. This latter step is competitively inhibited by INSTIs.

INSTIs that are currently available include raltegravir (RAL), elvitegravir (EVG), and dolutegravir (DTG). These antiretrovirals are considered effective, minimally toxic, and tolerable and are now recommended for the treatment of newly diagnosed individuals living with HIV by the U.S. Department of Health and Human Services. All of these drugs have demonstrated non-inferiority to efavirenz (EFV)-based regimens with respect to viral suppression [6-10]. In addition, the SAILING study demonstrated that DTG was superior to RAL at week 48 in patients infected with viruses resistant to two or more non-INSTI antiretroviral drug classes [11], suggesting a possible use for DTG in INSTI-naïve treatment-experienced individuals. RAL and EVG possess a modest genetic barrier to resistance which threatens the long-term use and efficacy of these INSTIs in patients with poor adherence to therapy. In particular, mutational pathways associated with mutations at positions Y143, N155, and Q148 are associated with resistance to both RAL and EVG [12,13]. Mutations at position Q148 also reduce HIV susceptibility to the more recent INSTI DTG when Q148 substitutions are associated with several secondary mutations [14,15]. This newly approved INSTI [16] possesses a greater barrier to resistance and no major resistance mutation has yet been identified in treatment-naïve patients treated with DTG [10,15,17]. However, the R263K mutation has been found in INSTI-naïve ART-experienced patients receiving DTG treatment who have failed therapy with this drug [18]. As such, tissue culture selection studies with this drug have revealed the emergence of the R263K mutation in the integrase of HIV subtypes B and circulating recombinant form CRF02_A/G viruses [19]. R263K has been shown to confer low levels of resistance to DTG while decreasing viral fitness [19]. The same selection study also revealed multiple secondary mutations including the M50I polymorphism in subtype B integrase [19].

In previous studies, the M50I polymorphism has been found in 10-25% of INSTI-naïve patients [20]. In addition, this polymorphism has been observed in combination with R263K in a patient who subsequently failed treatment with RAL [21]. As a result, it is reasonable to speculate that the presence of M50I could compromise DTG activity in INSTI-naïve patients as well as contribute to cross-resistance in treatment-experienced patients. Here, we present the biochemical characterization of the M50I substitution alone and in combination with the primary resistance mutation R263K in subtype B integrase. We investigated the effect of these mutations on strand-transfer activity, viral fitness and resistance to INSTIs DTG, RAL, and EVG. Our results demonstrate that the M50I polymorphism does not restore the loss in HIV-1 infectivity associated with R263K and confers moderate resistance to DTG when combined with the latter resistance mutation.

## Results

### Occurrence of the M50I polymorphism in treatment-naïve individuals living with HIV-1 subtype B

**Figure 1:**
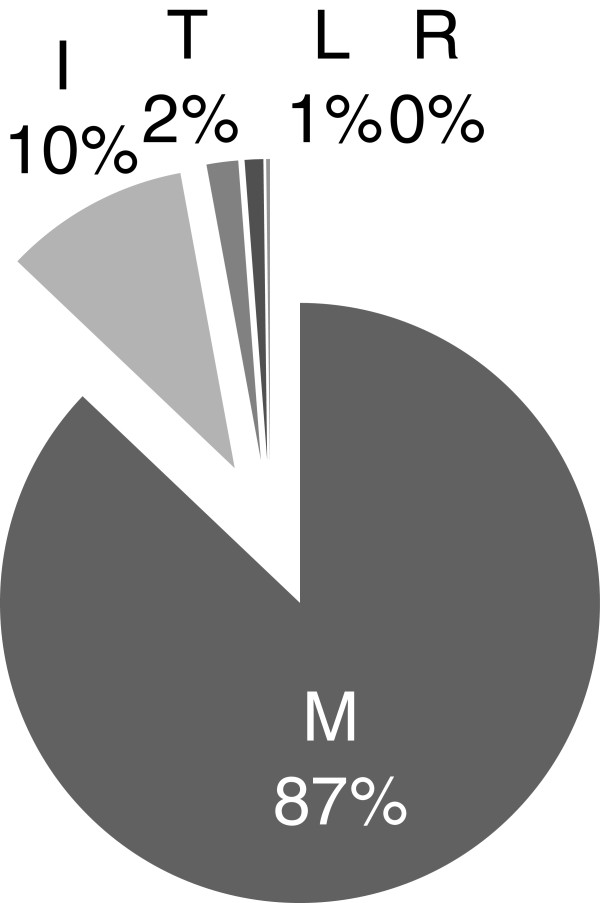
**Occurrence of the M50I polymorphism in treatment-naïve individuals living with HIV-1 subtype B.** Sequence analysis of subtype B integrase of 2,253 clinical isolates from treatment-naïve patients from the Stanford HIV Drug Resistance Database for the following polymorphisms: M50M, M50I, M50T, M50L and M50R.

The M50I polymorphism was selected in culture as a secondary mutation to R263K [19] as well as sequenced from a patient failing treatment with RAL [21]. As a result, we wanted to determine the frequency with which the M50I polymorphism occurs in the INSTI-naïve patient population. Analysis of clinical isolates of INSTI-naïve patients from the Stanford HIV Drug Resistance Database revealed that the M50I polymorphism was present at a frequency of 10% in subtype B integrase (Figure 1). The wild-type M50 was the most common genotype with a frequency of 87% suggesting that integrase subtype B is not highly variable at this position.

### Addition of M50I to R263K does not increase integrase strand-transfer activity

**Figure 2:**
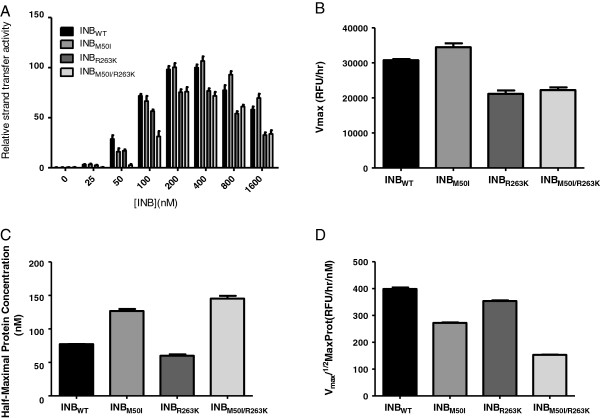
**Addition of M50I to R263K does not increase integrase strand-transfer activity.** Strand-transfer activity and *V*_max_/_1/2_MaxProt of wild-type and mutant enzymes. **A)** Relative strand-transfer activity when varying protein concentration. **B)***V*_max_ values. **C)**_1/2_MaxProt values. **D)** Enzyme efficiency as determined by the division of *V*_max_ by _1/2_MaxProt. Error bars represent the standard errors of the means (SEM).

**Figure 3:**
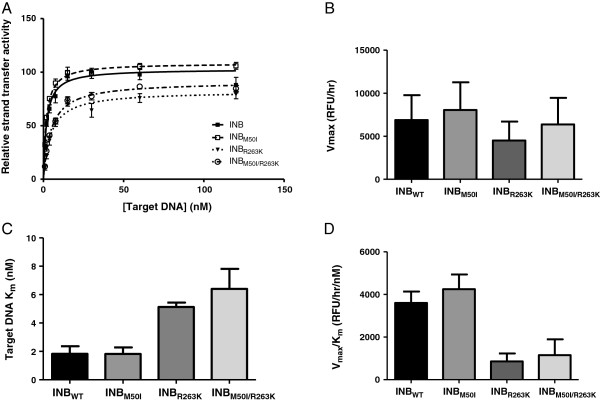
**The effect of M50I with R263K on enzyme activity.** Strand-transfer activity and enzyme efficiency of wild-type and mutant enzymes. **A)** Relative strand-transfer activity when varying the concentration of biotinylated target DNA. **B)***V*_max_ values. **C)***K*_m_ values. **D)** Enzyme efficiency as determined by the division of *V*_max_ by *K*_m_. Error bars represent the standard errors of the means (SEM).

Previous studies have demonstrated that the primary resistance mutation R263K decreases integrase activity in cell-free assays [19]. Here, with the use of wild-type and mutant integrase proteins, we show that M50I does not compensate for the decrease in enzymatic activity associated with R263K. When varying the concentration of the INB_WT_, INB_M50I_, INB_R263K_, and INB_M50I/R263K_ proteins (Figure 2A), the activity of the M50I mutant initially appeared to be greater than that of the WT enzyme (Figure 2B). However, further investigation revealed that the *V*_max_/_1/2_MaxProt values of the M50I and R263K enzymes were decreased compared to WT (Figures 2C-D). The addition of M50I to R263K did not compensate for the decreased *V*_max_/_1/2_MaxProt of the R263K enzyme (Figure 2D). The results were similar when we varied the concentration of target DNA (Figure 3). In these experiments, both the *V*_max_ and *K*_m_ of the M50I enzyme were comparable to those of the WT enzyme whereas R263K resulted in a decreased *V*_max_ and increased *K*_m_ (Figures 3B-D). The addition of M50I to R263K slightly increased integrase *V*_max_ but further increased the *K*_m_, resulting in a non-significant increase in enzyme efficiency (Figure 3D). Overall, the addition of M50I to R263K did not compensate for the decrease in enzymatic efficiency associated with the R263K mutant.

### The effect of M50I with R263K on susceptibility to INSTIs

The integrase coding region of HIV-1 contains similar levels of natural variation as that seen in protease [22]. Furthermore, the pre-existing polymorphism L63P which emerges during treatment with PIs has been shown to be compensatory when combined with a primary resistance mutation [22]. We therefore wanted to determine if the addition of M50I, a natural polymorphism in integrase, to R263K affected susceptibility to DTG, RAL, and EVG in strand-transfer assays. Analysis with the competitive inhibition model was used to generate values of relative *V*_max_ and *K*_i_ expressed in fold-change (FC) (Table 1). The R263K mutation did not greatly affect DTG activity (FC = 2.6) and the addition of M50I did not have a significant effect on the level of resistance observed (FC = 2.8). In contrast, the addition of M50I to R263K decreased susceptibility to EVG (FC = 6.4 for M50I/R263K compared to 3.0 for R263K alone) and RAL (FC 5.4 compared to 4.2). The M50I mutant enzyme was associated with low-level resistance to all INSTIs tested (FC ~ 2.5) in these cell-free assays.

**Table 1:** **Effects of the M50I and R263K mutations on DTG, RAL, and EVG inhibitory constants (*K***_**i**_**)**

	**DTG**		**RAL**		**EVG**	
**INB**	**Relative**	**Fold**	**Relative**	**Fold**	**Relative**	**Fold**
	***V***_**max**_	**Change (*K***_**i**_**)**	***V***_**max**_	**Change (*K***_**i**_**)**	***V***_**max**_	**Change (*K***_**i**_**)**
WT	100	1	100	1	100	1
M50I	109	2.085	118	2.467	108	2.2
R263K	105	2.627	106	5.404	117	6.4
M50I/R263K	108	2.824	95	4.255	120	3

Using TZM-bl assays with pNL4.3 M50I and R263K viruses, we also showed that the combination of M50I and R263K increased resistance to DTG (FC = 15.6 fold) compared to R263K alone (FC = 8.5 fold) (Table 2). When these experiments were repeated with EVG, the R263K mutation alone conferred moderate-level resistance (FC = 21.4 fold) and, when combined with M50I, resistance to EVG was further increased (FC = 34.4 fold). In contrast, the M50I/R263K double mutant conferred only low-level resistance to RAL (FC = 3.6 fold). M50I alone did not confer resistance to DTG or RAL but did confer low-level resistance to EVG (FC = 5.4 fold).

**Table 2:** **Effects of the M50I and R263K mutations on IC**_**50**_**s for DTG, RAL, and EVG**

		**DTG**		**RAL**		**EVG**	
**Backbone**	**Genotype**	**IC**_**50 (nM)**_	**FC**	**IC**_**50 (nM)**_	**FC**	**IC**_**50 (nM)**_	**FC**
pNL4.3	WT	0.3113	**-**	0.1023	-	1.082	-
	M50I	0.6053	1.94	0.04851	0.47	5.9	5.45
	R263K	2.662	8.55	0.1898	1.85	23.16	21.4
	M50I/R263K	4.854	15.59	0.3643	3.56	37.26	34.44

### M50I does not compensate for the reduction in HIV replication associated with R263K

**Figure 4:**
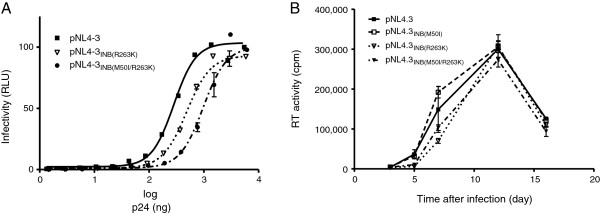
**M50I does not compensate for the reduction in HIV replication associated with R263K.** Effects of the M50I and R263K mutations on HIV infectivity in TZM-bl cells **(A)** and replication capacity in PM1 cells **(B)**.

To determine whether M50I might impact viral replication capacity, we performed TZM-bl infection assays with varying amounts of wild-type pNL4.3, pNL4.3_INB(R263K)_, and pNL4.3_INB(M50I/R263K)_ viruses (Figure 4). As previously demonstrated [19,23] and confirmed here, the R263K single mutation modestly diminished HIV infectivity whilst the addition of M50I to R263K further increased this deficit (Figure 4A). Long-term infection studies confirmed these results although the R263K replication deficit was mostly observed early in the infection course (Figure 4B). The M50I mutant alone did not negatively impact HIV replication capacity; however, the addition of M50I to R263K further decreased viral fitness. Combined with our biochemical results, these data indicate that the M50I mutation does not compensate for the loss in replication fitness conferred by R263K.

## Discussion

M50I is an accessory mutation that was selected in tissue culture subsequent to the emergence of R263K under DTG pressure [23]. Furthermore, this natural polymorphism has been detected in clinical isolates [21] and can be found in 10-25% of INSTI treatment-naïve patients [20]. Primary mutations, such as R263K, can often negatively impact integrase enzymatic activity and lower viral replication capacity [24]. Secondary mutations therefore compensate for this by increasing levels of drug resistance while simultaneously restoring viral fitness [24]. Here, we provide evidence that M50I alone does not negatively impact integrase strand-transfer activity and HIV replication capacity, an observation that explains the existence of this polymorphic substitution in untreated patients.

In addition to M50I, other secondary mutations have emerged in the presence of R263K in tissue culture experiments with DTG, i.e. E138K and H51Y [19]. This latter mutation has previously been characterized [23]. Comparable to H51Y, the addition of M50I to R263K increased resistance against DTG (15.6-fold for M50I/R263K versus 16.5-fold for H51Y/R263K). Furthermore, both of the H51Y and M50I mutations were innocuous when tested in the absence of R263K. In contrast, the addition of H51Y to R263K was very detrimental to integrase strand-transfer activity [23]. Although the combination of M50I/R263K did not restore enzyme efficiency (Figure 3D), the M50I polymorphism partially restored integrase maximal activity to 92% of the WT *V*_max_ for M50I/R263K (Figure 3B), as compared to approximately 20% for H51Y/R263K [21]. Since the M50I/R263K double mutant decreased integrase affinity for DNA compared to R263K alone (Figure 3C), an effect also observed with H51Y [23], this combination of mutations did not restore integration. In agreement with these results, the addition of M50I to R263K did not restore viral infectivity and replication capacity, similar to what has been reported for H51Y/R263K [23]. These results are in accordance with previous results regarding polymorphisms and their role in conferring drug resistance, i.e. patient-derived viruses with polymorphisms do not demonstrate a decrease in susceptibility to INSTIs unless a primary resistance mutation is also present [22,25].

Although innocuous for RAL and DTG, the presence of M50I may slightly decrease HIV-1 susceptibility to EVG. The clinical relevance of this finding will require further investigation. In particular, it should be investigated whether M50I is over-represented in patients who have failed EVG-based therapy in clinical trials [8,26-28]. Overall, M50I by itself is unlikely to lead to treatment failure. Besides its effect on susceptibility to EVG, the biochemical characterization of the M50I integrase enzyme demonstrates that this polymorphism alone does not affect strand-transfer activity. This observation is in agreement with the natural prevalence of this substitution since integration is essential to HIV-1 infectivity. When associated with the R263K mutation, M50I demonstrates a tendency toward improving maximal strand-transfer activity while decreasing integrase affinity for target DNA, as measured by *V*_max_ and *K*_m_ values, respectively (Figure 3). This strongly suggests that M50I is unable to compensate for the R263K defect in DNA binding that has been previously reported [19].

## Conclusion

The addition of M50I to R263K fails to restore the deficit in HIV replication capacity conferred by R263K. This observation is in agreement with the hypothesis that the R263K resistance pathway may represent an evolutionary dead-end for HIV-1; however this hypothesis requires further evaluation and remains to be proven. These results also help to explain the absence of *de novo* resistance mutations in treatment-naïve patients who have been treated with DTG in clinical trials.

## Methods

### Cells and antiviral compounds

Infectivity experiments were performed using TZM-bl cells and 293T cells for purposes of transfection with replication-competent wild-type or mutant HIV-1. TZM-bl cells were obtained from the NIH AIDS Research Reagent Program. The 293T cell line was obtained from the American Type Culture Collection (CRL-11268). Both cell lines were subcultured every 3–4 days in Dulbecco’s minimal essential medium (DMEM) and kept at 37°C under 5% CO_2_. DTG, RAL, and EVG were provided by GlaxoSmithKline/ViiV Healthcare, Merck Inc, and Gilead Sciences, respectively.

### Protein expression and purification

Wild-type and mutant subtype B integrase proteins were expressed using *Escherichia coli* BL21 (DE3) Gold cells, F_ *ompT hsdS*B(rB_ mB_) *dcm gal* _(DE3)(Stratagene). Bacterial cultures were grown at 37°C in 500 ml Luria-Bertani (LB) broth supplemented with 100 μg/ml ampicillin until they reached an optical density of 0.4-0.6 at 600 nm. Protein expression was induced with 1 mM isopropyl-b-D-thiogalactopyranoside (IPTG) for 3 hr at 37°C, 200 rpm. The cultures were then centrifuged at 7,000 rpm for 10 min. The cell pellets were stored at -80°C. The purification of integrase recombinant proteins was performed as previously described for His-tagged integrase [19,29].

### Integrase strand-transfer activity

The strand-transfer activity of the wild-type, M50I, R263K, and M50I/R263K integrase subtype B proteins were measured using a microtiter plate assay as previously described [19]. Briefly, equimolar amounts of donor DNA LTR sense, 5′AmMC12ACCCTTTTAGTCAGTGTGGAAAATCTCTAGCAGT-3′, and antisense, 5′-ACTGCTAGAGATTTTCCACACTGACTAAAAG-3′, were annealed by heating for 10 min at 95°C. Once cooled, the DNA LTR functional duplexes were covalently linked to Costar DNA-Bind 96-well plates (Corning) and stored at 4°C. After 48 hr, the plates were blocked and washed as previously described [19]. The wild-type and mutant integrase proteins were diluted to a final concentration of 400 nM and incubated on the plates at room temperature for 30 min. Next, the indicated concentrations of INSTIs were either added or not, followed by the addition of the biotinylated target DNA duplex (sense 5′-TGACCAAGGGCTAATTCACT-3Bio, and antisense (D), 5′-AGTGAATTAGCCCTTGGTCA-3Bio). Following incubation for 1 hr at 37°C, the plates were washed twice and quantified with Eu-labelled Streptavidin (PerkinElmer) as previously described [19].

### Generation of replication-competent HIV-1

The pNL4.3_INB(R263K)_ plasmid has been described previously [19]. The pNL4.3_INB(M50I)_ and pNL4.3_INB(M50I/R263K)_ replication-competent HIV-1 plasmids were generated using the QuickChange II XL Site-Directed mutagenesis kit. The primers used for the M50I mutation were: sense, 5′-CTAAAAGGGGAAGCCATACATGGACAAGTAGACTG-3′ and antisense, 5′-CAGTCTACTTGTCCATGTATGGCTTCCCCTTTTAG-3′. Following mutagenesis, the plasmids were digested with Dpn1 for 4 hr at 37°C and transformed using *Escherichia coli* strain XL10-Gold ultracompetent cells, Tetr _(*mcrA*)*183* _(*mcrCB-hsdSMR-mrr*)*173endA1 supE44 thi-1 recA1 gyrA96 relA1 lac* Hte [F = *proAB lacI*q*Z*_M15Tn*10* (Tetr) Amy Camr] (Stratagene). The QIAprep MiniPrep Kit (QIAGEN) was used for plasmid purification and the plasmids were quantified with NanoDrop. Presence of the mutations was confirmed by sequencing. Genetically homogenous HIV-1 viruses were produced by transfecting 12.5 μg of wild-type or mutant pNL4.3 plasmids into 293T cells as previously described [19]. An enzyme-linked immunosorbent assay (ELISA) (ABL, Inc.) was used to measure levels of p24 in culture. Reverse Transcriptase (RT) activity was measured as previously described [30].

### Resistance assays in TZM-bl cells

HIV susceptibility to DTG, RAL, and EVG was determined using short-term resistance assays with TZM-bl cells as previously described [19]. Briefly, 30,000 cells per well were infected with the WT, M50I, R263K, or M50I/R263K viruses in the presence of serial dilutions of DTG, RAL, or EVG in 96-well plates (Corning). The amount of virus added to each well was normalized using results from RT activity. After incubation for 48 hr at 37°C and 5% CO_2,_ luciferase activity was measured using the luciferase assay system (Promega) and a Micro-Beta2 luminometer (PerkinElmer).

### HIV infectivity and replication capacity

Non-competitive short-term infectivity assays in TZM-bl cells were used to evaluate HIV infectivity as previously described [19]. Long-term infection assays were used to determine HIV replication capacity in PM1 cells by quantifying RT activity (cpm) as previously described [23,30].

### Data analysis

Unless otherwise indicated, all experiments consisted of at least 2 sets of experiments performed in triplicates, yielding 6 independent values for each data point. For each experiment, strand-transfer values in the absence of drug were arbitrarily determined as 100%. When varying concentrations of target DNA and protein were employed, strand-transfer results were fit to the Michaelis-Menten equation with the use of GraphPad Prism 5.0 software to generate values for *V*_max_, *K*_m_ and *K*_i._ The half-maximal protein concentration, defined as ½ of the calculated concentration at which maximal activity is reached, was determined at varying INB concentrations. Enzyme performance was determined by dividing *V*_max_ by *K*_m_ as previously described [31]. The strand-transfer activity of the wild-type and mutant enzymes in the presence of INSTIs was determined using the competitive inhibition model and by constraining to *K*_m_ values based on the delta target DNA results. The *K*_m_ values for the INB_WT_, INB_M50I_, INB_R263K_, INB_M50I/R263K_ enzymes were 3.745, 8.997, 11.40 and 9.660, respectively. Using replication capacity experiments in TZM-bl cells in the presence of DTG, RAL or EVG, fifty percent inhibitory concentrations (IC_50_s) were determined for the wild-type and mutant viruses using the sigmoid dose–response function of the same software.

## Competing interests

The authors declare no competing financial interests.

## Authors’ contributions

MW designed and performed experiments, analysed data and wrote the manuscript; TM designed and performed experiments, analysed data and revised the manuscript; NO, PKQ, and YH performed experiments; and MAW supervised the project and revised the manuscript. All authors read and approved the final manuscript.
